# Anterior Cervical Discectomy and Fusion With “Kissing” Allograft Interbodies

**DOI:** 10.7759/cureus.19499

**Published:** 2021-11-12

**Authors:** Jonathan Rasouli, Brian Fiani, John Belding, Timothy A Moore

**Affiliations:** 1 Neurosurgery, Cleveland Clinic, Cleveland, USA; 2 Neurosurgery, Desert Regional Medical Center, Palm Springs, USA; 3 Orthopaedic Surgery, MetroHealth Medical Center, Cleveland, USA

**Keywords:** interbody, allograft, fusion, disc degeneration, cervical spine

## Abstract

Background: There is recent evidence to suggest that the use of polyetheretherketone (PEEK) interbodies are inherently associated with a higher rate of pseudarthrosis, in particular, at the C5-6 and C6-7 levels. Herein, we describe our technique utilizing two parallel structural allografts or “kissing” allografts, designed to mitigate the risk of pseudarthrosis and subsidence at these levels.

Materials and Methods: We retrospectively reviewed all anterior cervical discectomy and fusion (ACDF) procedures with “kissing” for degenerative spine pathology at a single institution between 2018 and 2019 for the C5-6 and C6-7 levels. One-year postoperative flexion/extension cervical X-rays were evaluated for evidence of radiographic pseudarthrosis and subsidence.

Results: A total of 28 patients met the study criteria. Solid fusion was achieved in 93%. There were no infections or wound complications. One patient developed postoperative dysphagia that resolved at 3-months post-op. Two patients were found to have clinically asymptomatic radiographic pseudarthrosis that did not warrant intervention. One patient developed a postoperative hematoma that required surgical evacuation.

Conclusions: “Kissing” allograft ACDF is a safe and effective method designed to address the intrinsically higher risk of pseudarthrosis at the C5-6 and C6-7 levels. Further prospective studies are warranted to comparatively evaluate this technique against single allograft and PEEK interbodies.

## Introduction

Anterior cervical discectomy and interbody fusion (ACDF) for degenerative cervical pathology is a common inpatient surgery performed in the United States [1]. First described in the mid-20^th^ century by pioneers such as Smith, Robinson, and Cloward, anterior approaches have rapidly gained in popularity due to their effectiveness at treating degenerative disc pathologies with minimal complications, high fusion rates, reduced patient recovery time, and allowing direct treatment of cervical kyphotic deformities [2-4]. Over time, spinal instrumentation and techniques have evolved; however, pseudarthrosis and graft subsidence remain a persistent challenge [3].

There remains controversy regarding the reported incidence of radiographic and clinical pseudarthrosis with the use of polyetheretherketone (PEEK) interbodies after ACDF [5-8]. Furthermore, there is evidence to suggest that the C5-6 and C6-7 levels have an intrinsically higher risk of pseudarthrosis than other cervical levels [9]. While approximately 30% of patients with pseudarthrosis are asymptomatic, several studies have demonstrated improvements in patient-reported outcomes with the formation of a solid fusion [5,10]. Therefore, new technologies, interbody options, and osteobiologic agents have been developed to enhance fusion rates. Unfortunately, these products can be cost-prohibitive and lack long-term evidence-based studies. The question exists as to whether existing interbody options and surgical techniques can be easily and inexpensively modified to enhance fusion rates after ACDF.

In this manuscript, we describe the “kissing” allograft technique that was developed to mitigate pseudarthrosis in ACDF procedures that specifically incorporate the C5-6 and C6-7 levels. We have used this technique to obviate several of the inherent challenges in obtaining a solid fusion at these levels when there is degenerative disc pathology. To date, this technique has been performed in 28 patients with satisfactory fusion rates and minimal post-operative complications.

## Materials and methods

We conducted a retrospective, observational study spanning January 2018 to December 2019 examining all patients who underwent elective single and multi-level ACDF with “kissing” allograft interbodies at a single institution. The interbodies are FDA approved and available in North America. Patient charts, operative notes, radiographs, and post-operative clinic notes were reviewed to evaluate fusion rates, complications, and clinical outcomes. Bony fusion was evaluated on lateral cervical x-rays obtained at one-year post-op, as previously described by Teton and colleagues [6]. Exclusion criteria included any previous anterior or posterior cervical fusion surgery. Institutional review board approval was granted for this study.

Technique description

After informed consent was obtained, the patient was taken to the operating room and positioned supine on the table. General endotracheal anesthesia was administered, and the endotracheal tube was taped contralateral to the desired surgical approach. The head was placed in a neutral or gently extended position on a foam donut headrest to optimize lordotic alignment. The anterior neck was then prepped and draped in the usual fashion.

**Figure 1:**
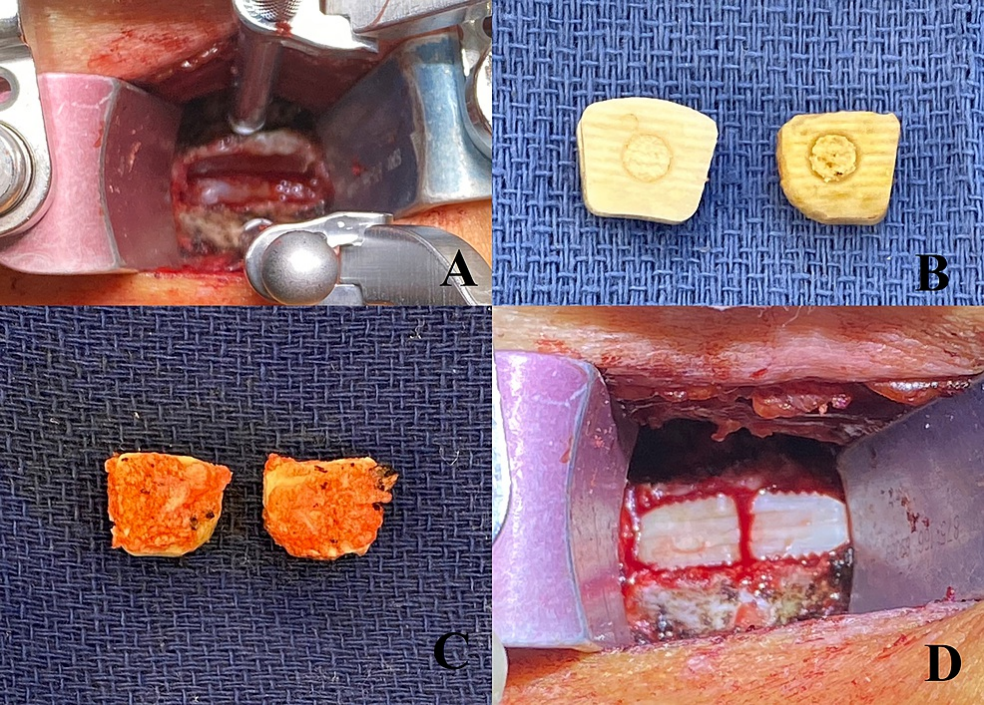
Proper intraoperative allograft interbody placement to create the “kissing” ACDF construct. (A) intraoperative image demonstrating a completed cervical discectomy with removal of the posterior longitudinal ligament. A trial spacer is inserted inside the discectomy defect to select an appropriate interbody height. In this case, a 7mm trial was chosen. (B) Two 7mm structural allografts are then circumferentially shaved with the high-speed electric drill in order to allow them to fit in a parallel orientation in the discectomy defect. The sample allograft on the left-hand of the image demonstrates the allograft prior to shaving. (C) The shaved allografts are filled with bone dust (or demineralized bone matrix) collected with the specimen trap and then gently tamped into the discectomy defect (D) Final intraoperative view of the “kissing” allografts.

**Figure 2:**
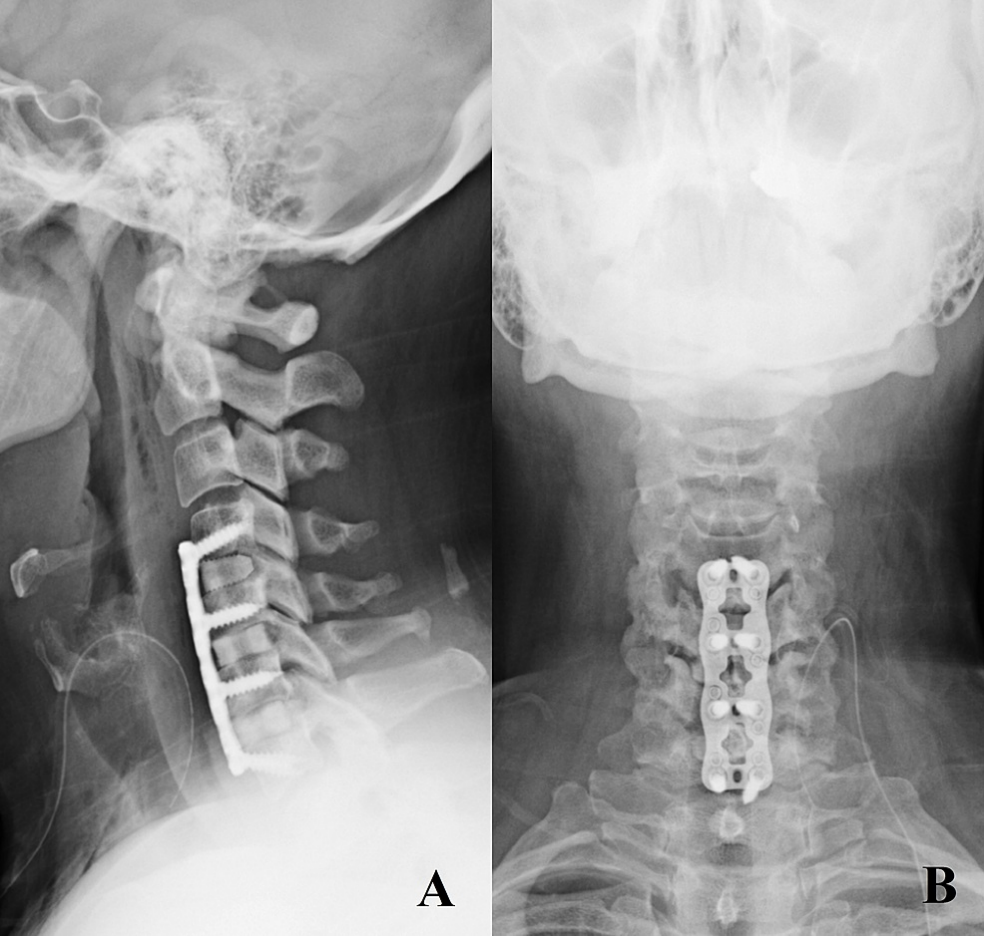
Postoperative x-ray after C4-7 ACDF with “kissing” allografts at C5-6, C6-7. (A) lateral and (B) AP views demonstrating prior graft placement on postoperative day one. A single allograft interbody was used at C4-5.

**Figure 3:**
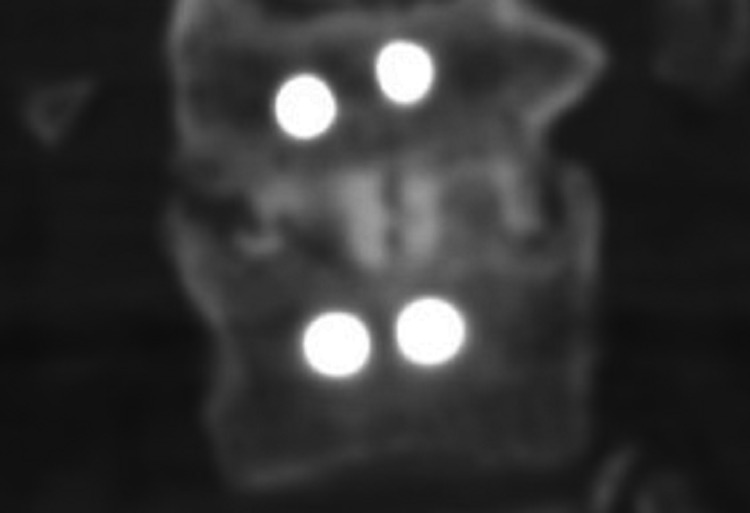
Post-operative computerized tomography scan after C5-6 ACDF demonstrating solid arthrodesis.

A transverse incision was created in a prominent skin crease, and a standard Smith-Robinson ACDF approach was performed [4]. Once the C5-6 or C6-7 disc space was encountered and confirmed with intraoperative x-ray, Caspar pin distraction was accomplished across the disc space. A complete discectomy from the uncovertebral joint to the uncovertebral joint was carried out with curettes, pituitary rongeurs, 4-mm round cutting burr attached to a high-speed electric drill, and a 2-mm Kerrison. Local bone shavings were collected in a 40cc specimen trap connected to suction (Busse, Hauppauge, NY, USA). The posterior longitudinal ligament was removed. The discectomy defects were measured with a trial spacer, and then two matching lordotic corticocancellous allografts (12 mm x 14 mm) (Globus Medical, Audubon, PA, USA) were brought to the operating table and gently fashioned with the cutting burr to allow a snug fit into the disc space. Local autograft was collected from the disc space, preparation was mixed into the interbodies, and then tamped into place (Figure 1). Caspar pin distraction was released. A rigid titanium plate was affixed to the anterior vertebral body with careful attention to remove osteophytes before placement. Variable vertebral body screws were inserted at all slots. A final x-ray was obtained, and the wound was closed in layers. A Penrose or closed-suction drain was placed for all multi-level or revision ACDF procedures. Postoperative X-rays show the ideal placement of construct and identify the interbodies as described in Figure 2. Additionally, a postoperative CT scan identifies successful arthrosis of the cervical disc space (Figure 3).

## Results

A total of 28 patients met the study criteria. 64% (n=18) were male. Indications for surgery included 71% (n=21) degenerative cervical spondylosis with myelopathy with or without radiculopathy and 29% (n=7) unilateral or bilateral medically refractory cervical radiculopathy. Eleven (39%) patients underwent single-level ACDF (C5-6 (n=3), C6-7 (n=8)). Nine (32%) patients underwent C5-7 ACDF. Eight patients (28%) underwent C4-7 ACDF. There were no instances of post-operative wound infections, wound complications, esophageal injury, or hoarseness. A solid fusion, as determined by one-year postoperative flexion/extension cervical x-rays, was achieved in 93% of patients. One patient developed postoperative dysphagia that resolved at three-months post-op. Two patients had evidence of radiographic pseudarthrosis on plain films, without clinical symptoms, that did not warrant intervention. One patient developed a postoperative hematoma that required surgical evacuation. Unfortunately, the patient-reported outcome instruments were not available to obtain objective measurements of postoperative clinical progress. However, based on the descriptive post-operative clinic notes, the majority (89%, n=25) of patients had satisfactory to good clinical outcomes at one year.

## Discussion

With the rise of allograft and xenograft interbody options over structural iliac crest bone graft, there has been controversy regarding the incidence of pseudarthrosis and graft subsidence after ACDF [5-7,9]. In a systematic review of cervical pseudarthrosis by Leven and Cho, there was an overall fusion rate of 96% with one level ACDF with plate, 80%-95% fusion rate with 2-level ACDF with plate, and 70-80% fusion rate with > 2 level ACDF with plate with the use of structural allograft [5]. Although there are purported advantages of using PEEK interbodies, there is evidence to suggest that they do not promote fusion as readily as allograft [6,11]. In a recent study by Teton and colleagues, there was a more than 6-fold incidence of radiographic pseudarthrosis with the use of PEEK interbodies for multilevel ACDF compared to structural allograft at one-year [6]. This is in contrast to a study performed Wang and colleagues who found there was no statistical difference in pseudarthrosis and subsidence rates between allograft and PEEK interbodies at two-year follow-up for single and multilevel ACDF [7]. However, the main limitations in these studies are their retrospective design, heterogeneity in the definition of pseudarthrosis, levels operated, and mean follow-up time.

Numerous perioperative techniques, interbody designs, and osteobiologic products have been described to mitigate pseudarthrosis rates after ACDF. The use of low-dose (0.7 mg/level) recombinant human bone morphogenic protein (rhBMP-2) has demonstrated excellent fusion rates; however, at the expense of a higher incidence of symptomatic dysphagia and seroma formation [11,12]. These complications can be reduced if the rhBMP-2 sponge is placed exclusively inside the interbody and/or using ultra-low-dose (0.25 mg/level) concentrations [13]. i-Factor™ Bone Graft (Cerapedics Inc, Westminster, CO, USA), a composite bone substitute consisting of synthetic P-15 protein that recruits osteogenic cells, has also demonstrated better fusion rates with minimal complications compared to allograft for single-level ACDF for cervical radiculopathy at two years [14]. Porous titanium and titanium-coated PEEK interbodies are recent products that have also shown potential in reducing pseudarthrosis rates in animal models by improving the surface area of the bone-implant interface [15].

Despite these innovative technologies, pseudarthrosis rates at C5-6 and C6-7 remain persistently stubborn. This was demonstrated in a study by Van Eck and colleagues that examined the revision rate and occurrence of adjacent segment disease and pseudarthrosis in a cohort of 672 patients who underwent ACDF [9]. In this study, 92% of all pseudarthrosis occurred at these levels (39% C5-6, 53% C6-7), and the overall revision rate was 15% [9]. The question remains as to whether using PEEK interbodies at C5-6 and C6-7 in multi-level ACDF constructs is a prudent choice, given recent evidence of their inherently higher risk of pseudarthrosis [6].

**Figure 4:**
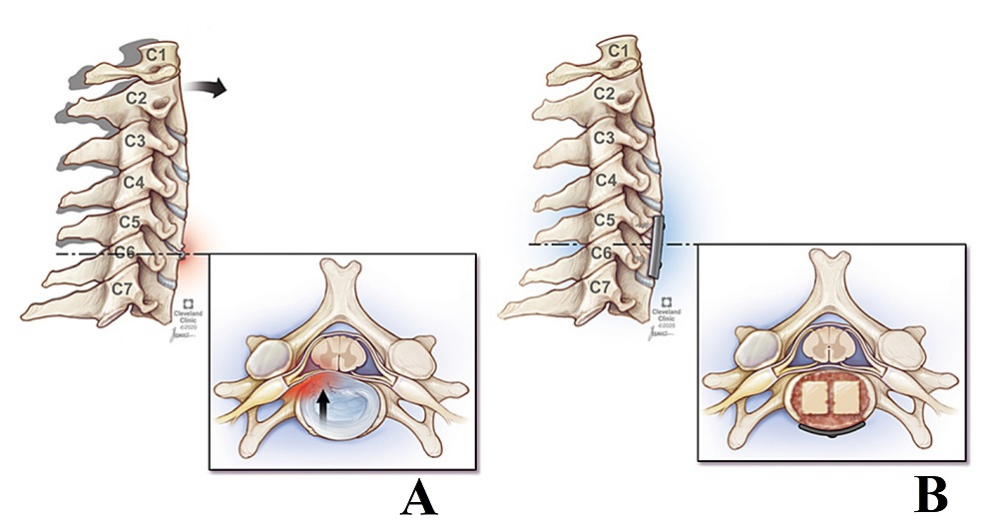
“Kissing” allograft anterior cervical discectomy and fusion (A) Illustrative example of degenerative disc disease at C5-6 with a right-sided paracentral disc bulge (B) C5-6 after anterior cervical discectomy, placement of allograft interbodies, demineralized bone matrix, and plate.

Our experience suggests that the use of structural allografts at C5-6 and C6-7 may improve fusion rates in single and multi-level ACDFs that incorporate these levels (Figure 4). The “kissing” allograft technique was developed, in part, to improve on standard structural allograft by creating double the surface area for fusion and more contact points for bony over-growth and in-growth. In terms of cost-effectiveness, Virk and colleagues found that allograft interbodies provide a more cost-effective quality-adjusted life year compared to PEEK ($2492 vs. $3328) [16].

The main limitation of this technique is the need to shave the allograft interbodies with the electric drill in order to allow them to fit snugly within the discectomy space and the need for two allografts. Theoretically, iliac crest bone graft (ICBG) could be used instead of an allograft; however, there are inherent complications with harvesting iliac crest, including significant donor site pain and hematomas [17]. In addition, Samartzis and colleagues found no difference in fusion rates between the use of allograft vs. ICBG in two separate retrospective studies [18,19]. Therefore, we prefer to use structural allograft instead of ICBG. Limitations to our study include its retrospective design, lack of patient-reported outcome instruments to gauge pre- and post-operative clinical outcomes, and nonrandomization of patients, which could theoretically introduce the risk of selection bias. We preferred to focus on the technical aspects of the procedure as well as the radiographic fusion rates, given the heterogeneity of patient outcome reporting when patient-reported outcome instruments are not utilized.

## Conclusions

In this article, we describe our “kissing” allograft technique that was designed to address an observed increase in our rate of pseudarthrosis with the use of single allograft and PEEK interbodies at the C5-6 and C6-7 levels. The main advantages are its higher surface area for fusion and more sites for autograft/allograft packing compared to the use of a single allograft interbody. Since we have implemented this technique, we have not observed any incidences of radiographic or clinical pseudarthrosis at these levels. Further prospective studies are warranted to evaluate comparative differences in fusion rates with “kissing” allografts versus single allografts and PEEK. 
